# Yellow Fever Microbe

**Published:** 1888-09

**Authors:** 


### YELLOW FEVER MICROBE.

The following letter addressed to Dr. J. McF. Gaston, of Atlanta, reflects somewhat severely on Dr. Sternberg, the medical gentleman sent out by our Government to inquire into the alleged discovery, by Dr. Freire, of Rio Janeiro, of the yellow fever microbe. We confess that the reprot of Dr. Sternberg did not impress us favorably, in that it seemed too hasty to allow time for a careful and thorough investigation of so important a matter. We append also some remarks by Dr. Gaston on the subject.

### DR. FREIRE'S LETTER.

RIO JANERIO, June 26, 1888.

Dear colleague and friend, I regret to inform you that Dr. Sternberg has published a report inimical to my labors. The wrong he thus inflicts on humanity is incalculable. I must tell you a few incidents relating to this subject. This gentleman did not stay in Brazil but about a month and a half, at a time when there was no epidemic. He did not see but one sporadic case of yellow fever. He made no autopsy. I now ask you if, on such bases, one can equip himself, even in a mediocre way, to assault works that have cost me so much toil and fatigue during more than eight years. You are well aware of the antipathy that Dr. Sternberg has always manifested to my discovery. I must answer him, and I am now preparing a refutation of his report.

This very year I have made an autopsy and found again the microbe in question. I am still inoculating with the most complete success. I hope you will continue aiding me in the task of resisting those men in their systematic plan of incredulity. The Government of your country made a bad appointment in delegating a physician whose ideas on this question were blinded by prejudice. I shall soon mail you a pamphlet containing a refutation to my adversaries and detractors.

I have too much courage to abandon the field, convinced as I am, of the truth of my investigations. One must struggle in order to conquer.

Accept, dear colleague, the assurance of my esteem and friendship.

DOMINGOS FREIRE.

To J. McF. Gaston, Atlanta, Ga.

### YELLOW FEVER INOCULATION.

In view of the grave consequences resulting from the prevalence of yellow fever at Jacksonville and the presumption raised in favor of the prevention or mitigation of this disease by inoculation with the attenuated virus, the occasion warrants an experiment in its application, which has been proved by a resort to its introduction in more than seven thousand subjects in Rio de Janeiro to be free from any risk to the life or health of the individual.

Dr. H. M. Lane, a former resident of Carthage, Mo., whose report has appeared in the medical journals of this country, gives the result of his own inoculation with the attenuated virus of the yellow fever during his sojourn in

Rio de Janeiro two years ago, indicating not only its freedom from any great disturbance of the vital functions, but its efficacy in securing the subject against an attack of the disease afterwards.

Whatever may be the differences of opinion as to the peculiar character of the microbe or germ of yellow fever, if it has been proven or even shown to be highly probable that inoculation with the virus in a modified form may afford exemption from so grave a malady, it would seem to be incumbent on the medical profession to test the virtues of this prophylactic procedure without regard to the explanations, which can be presented by bacteriologists of the mode in which the result comes about.

The commission undertaken by Dr. George M. Sternberg under the authority of the United States government during the past year, with a view to investigate the subject of yellow fever inoculation in Brazil and Mexico, was not attended with any satisfactory issues, owing to the fact of his visits being made to those countries at periods when the disease was not present in either, so as to afford any opportunity for the scientific or practical demonstrations of inoculation. His failure to verify what Domingos Freire claims to have discovered does not vitiate the main point to be settled, as to the prevention or mitigation of yellow fever by inoculation, and if he has not been provided with the attenuated virus by which the results may be tested in the subjects exposed to the disease in Jacksonville or elsewhere, it devolves upon him now to procure it and test this matter on a scale and under conditions which may enable our people to understand the true merits of this question, divested of all scientific speculation. The government has not met the requirements of humanity and common sense by authorizing a more scientific investigation of a matter involving the lives of her citizens to such an extent as the solution of this problem does, and if this application of yellow fever inoculation finds a barrier in official routine, then we appeal to municipal forecast to take such steps as may eliminate all doubt by a practical test of inoculation with the attenuatted virus of yellow fever.

The subject is too vast and too importaut to be disposed of in a few words, and I can only say that my opportunities for judging of the merits of this measure are of a nature that nothing short of an actual test of its efficacy, under the observation of competent observers, should satisfy the people of this country as to the claims in its favor made by the experiments of Domingos Freire in Rio de Janeiro. The recent fiasco of Dr. George Sternberg, who was commissioned by the United States government to investigate this subject, turns out to be a complete farce, and is not calculated to enlighten any one, touching the practical value of inoculation with the cultivated microbe of yellow fever in preventing or materially modifying the disease in those who are exposed to it. If he had gone to Tampa when the cases occurred there and applied the attenuated virus, so as to have judged of its effects, all could have had an opportunity of knowing what reliance was to be placed upon its use. He now has again a good field for testing inoculation among the people who remain in Jacksonville, and if he will draw on his practical resources by coming down to the plain matter of fact, the truth or the falsity of the claims for inoculation may be settled. —— J. McF. GASTON, M. D.

